# Supplementary material for: Cell-surface receptors enable perception of extracellular cytokinins
Source: Nat Commun. 2020 Aug 27;11:4284. doi: 10.1038/s41467-020-17700-9 (PMC7453015; doi:10.1038/s41467-020-17700-9)
Supplement: Supplementary file 1 — Supplementary Information [file 41467_2020_17700_MOESM1_ESM.pdf]

## Supplementary Information

# Cell-surface receptors enable perception of extracellular cytokinins


**Ioanna Antoniadi[1,2], Ondřej Novák[2,3], Zuzana Gelová[4,5], Alexander Johnson[4], Ondřej Plíhal[3,6,7], Radim Simerský[3,8], Václav Mik[3,6,], Thomas Vain[2,9], Eduardo Mateo-Bonmatí[2], Michal Karady[2,3], Markéta Pernisová[5], Lenka Plačková[3], Korawit Opassathian[1], Jan Hejátko[5], Stéphanie Robert[2], Jiří Friml[4], Karel Doležal[3,6], Karin Ljung[2,*] and Colin Turnbull[1,*†]**

[1] Department of Life Sciences, Imperial College London, London SW7 2AZ, United Kingdom.

[2] Umeå Plant Science Centre, Department of Forest Genetics and Plant Physiology, Swedish University of Agricultural Sciences, SE-901 83 Umeå, Sweden.

[3] Laboratory of Growth Regulators, Institute of Experimental Botany of the Czech Academy of Sciences and Faculty of Science of Palacký University, CZ-78371 Olomouc, Czech Republic.

[4] Institute of Science and Technology Austria, 3400 Klosterneuburg, Austria.

[5] CEITEC - Central European Institute of Technology and NCBR, Faculty of Science, Masaryk University, CZ-62500 Brno, Czech Republic.

[6] Department of Chemical Biology and Genetics, Centre of the Region Haná for Biotechnological and Agricultural Research, Faculty of Science of Palacký University, CZ-78371 Olomouc, Czech Republic.

[7] Department of Molecular Biology, Centre of the Region Haná for Biotechnological and Agricultural Research, Faculty of Science of Palacký University, CZ-78371 Olomouc, Czech Republic.

[8] Department of Protein Biochemistry and Proteomics, Centre of the Region Haná for Biotechnological and Agricultural Research, Faculty of Science of Palacký University, CZ-78371 Olomouc, Czech Republic.

[9] Present address: Chr-Hansen, 2630 Taastrup, Denmark *and* Department of Plant and Environmental Sciences, Copenhagen University, 2630 Tåstrup, Denmark

[*]Joint corresponding authors (emails: c.turnbull@imperial.ac.uk and karin.ljung@slu.se); [†]Lead Contact

## SUPPLEMENTARY TABLES

**Supplementary Table 1. Concentration of free cytokinin-linker ligand compounds and free cytokinin bases in medium after 16-hour incubation with immobilised cytokinins (*t*Z- or iP-type ligands attached to Sepharose beads, ligand mean density 5 µmol $L^{-1}$).** The experimental setup followed here was identical to the one in Fig. 4a, but without the addition of protoplasts, and included six washing steps of the beads prior to incubations. Incubations were done in duplicates, and both values are reported. Data indicate low leakage of free cytokinin bases (0.0004 – 0.0006%) and cytokinin-linker ligands (0.2 - 0.6%) derived from the compounds attached to Sepharose beads. When further correcting for the lower biological activity of the cytokinin-linker ligands, the total amounts of ligands detached from the beads would be insufficient to elicit the cytokinin signalling in Figures 3 and 4. Therefore, the signalling reported can be considered due to the immobilised cytokinins restricted to the extracellular space.

| | | **Cell culture medium incubated with immobilised cytokinins for 16 h** | |
|---|---|---|---|
| | | Concentration (nM) | |
| Compounds measured | | 0 h | 16 h |
| Cytokinin-linker ligands | *t*Z-linker ligand | 4.81 | 25.17<br>29.05 |
| | iP-linker ligand | 8.47 | 10.58<br>10.92 |
| Free cytokinins | *t*Z | 0.0031 | 0.0307<br>0.0300 |
| | iP | 0.018 | 0.018<br>0.019 |

**Supplementary Table 2. Quantification of iP and *t*Z levels in protoplast supernatant and pellet samples after 16 h of treatment with 2 µM iP.** Data indicate very low rates of turnover from iP (applied compound) into *t*Z, and therefore the effects on cytokinin signalling in Figures 3 and 4 can be considered results of the applied molecule (iP).

| | | **50 µl of protoplasts samples treated with 2 µM iP for 16 h** | | | |
|---|---|---|---|---|---|
| | | Levels of *t*Z and iP detected (fmol per 50 µL) | | % detected as *t*Z | |
| Fraction | | 8 h | 16 h | 8 h | 16 h |
| Supernatant | *t*Z | 5.44 ± 0.25 | 24.19 ± 0.6 | | |
| | iP | 1265.95 ± 52.92 | 1306.11 ± 11.64 | 0.43% | 1.85% |
| Pellet | *t*Z | 6.22 ± 0.41 | 16.74 ± 1.08 | | |
| | iP | 1573.12 ± 75.27 | 1268.92 ± 40.96 | 0.4% | 1.32% |

## SUPPLEMENTARY FIGURES

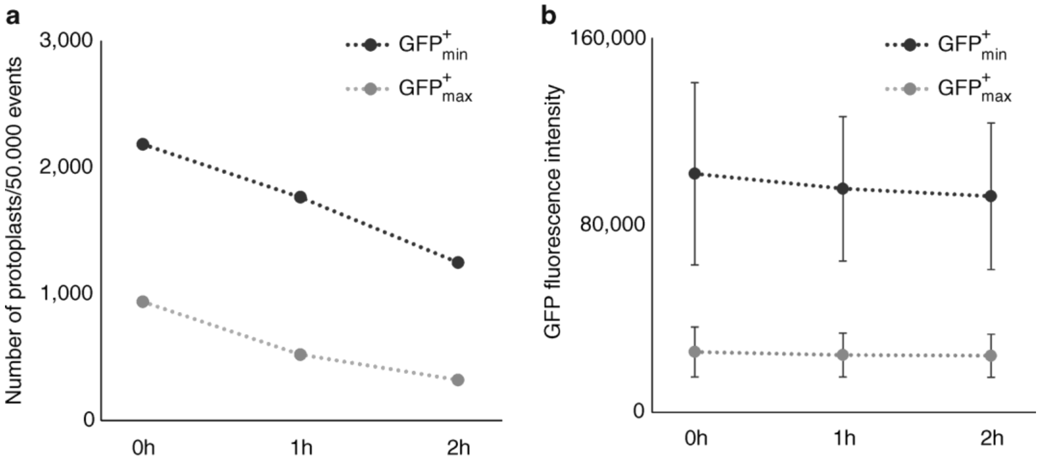


**Supplementary Figure 1. Control experiment testing GFP fluorescence stability over the 2 h of cell sorting.** *TCSn::GFP* root protoplasts were sorted into $GFP^{+}_{min}$ and $GFP^{+}_{max}$ cell populations as shown in Fig. 1c. At 0, 1 and 2 h, 50,000 events were recorded, and the $GFP^{+}_{min}$ and $GFP^{+}_{max}$ protoplast populations were examined for **a,** their number and **b,** their GFP fluorescence, as mean ± s.d. While there was a slight decrease in protoplast number, this is consistent in $GFP^{+}_{min}$ and $GFP^{+}_{max}$ cell populations, indicating that there is no mixing between the two groups during sorting. The high stability of GFP intensity during sorting and the respective significant difference between the two cell populations analysed are also shown.

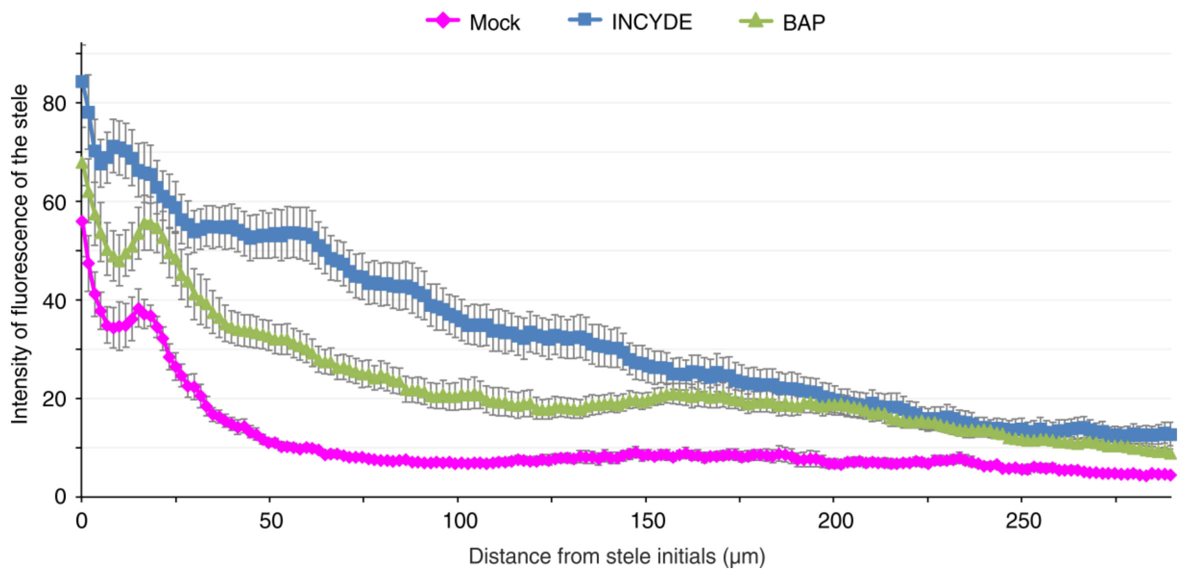


**Supplementary Figure 2. Responses of *TCSn::GFP*-expressing roots to treatment with exogenous cytokinin or INCYDE.** Quantification of the GFP fluorescence intensity in the stele of 6-day-old *TCSn::GFP* expressing roots using ImageJ. Seedlings were treated with the synthetic cytokinin 6-benzylaminopurine (BAP) (10 µM) or INCYDE (10 µM) for 6 h prior to confocal imaging. DMSO was used as mock treatment. n≥12 roots imaged per treatment, error bars are s.e.m.

**a**

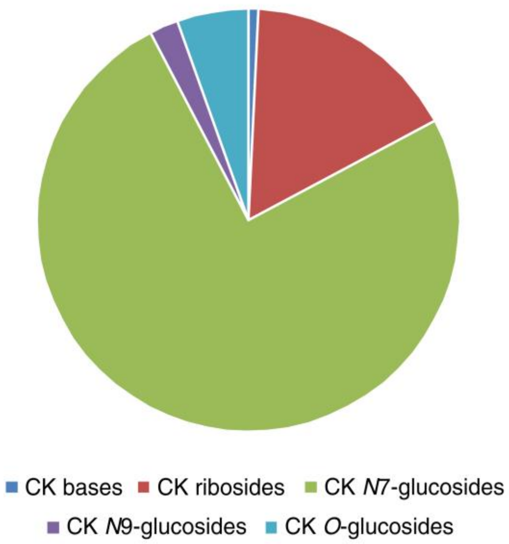


**b**

| | pmol/$10^5$ protoplasts |
|---|---|
| iP | 0.026 ± 0.003 |
| *t*Z | 0.091 ± 0.012 |
| *c*Z | 0.59 ± 0.05 |
| iPR | 0.59 ± 0.05 |
| *t*ZR | 0.27 ± 0.07 |
| *c*ZR | 14.58 ± 0.67 |
| DHZR | 0.009 ± 0.003 |
| iP7G | 62.71± 2.42 |
| *t*Z7G | 5.72 ± 0.36 |
| DHZ7G | 2.22 ± 0.09 |
| iP9G | 1.62 ± 0.14 |
| *t*Z9G | 0.15 ± 0.01 |
| *c*Z9G | 0.29 ± 0.06 |
| DHZ9G | 0.011 ± 0.002 |
| *t*ZOG | 2.54 ± 0.11 |
| *c*ZOG | 2.58 ± 0.34 |

**Supplementary Figure 3. Cytokinin compound distribution and absolute levels in root protoplasts.** Sum of cytokinin content in *TCSn::GFP*$^+$ and *TCSn::GFP*$^-$ root protoplasts, representing further processing of data in Fig. 2a-Mock. **a** Relative proportions of main classes of intracellular cytokinin compounds (% of total). **b** Absolute cytokinin quantification for individual compounds (fmol per 100,000 protoplasts).

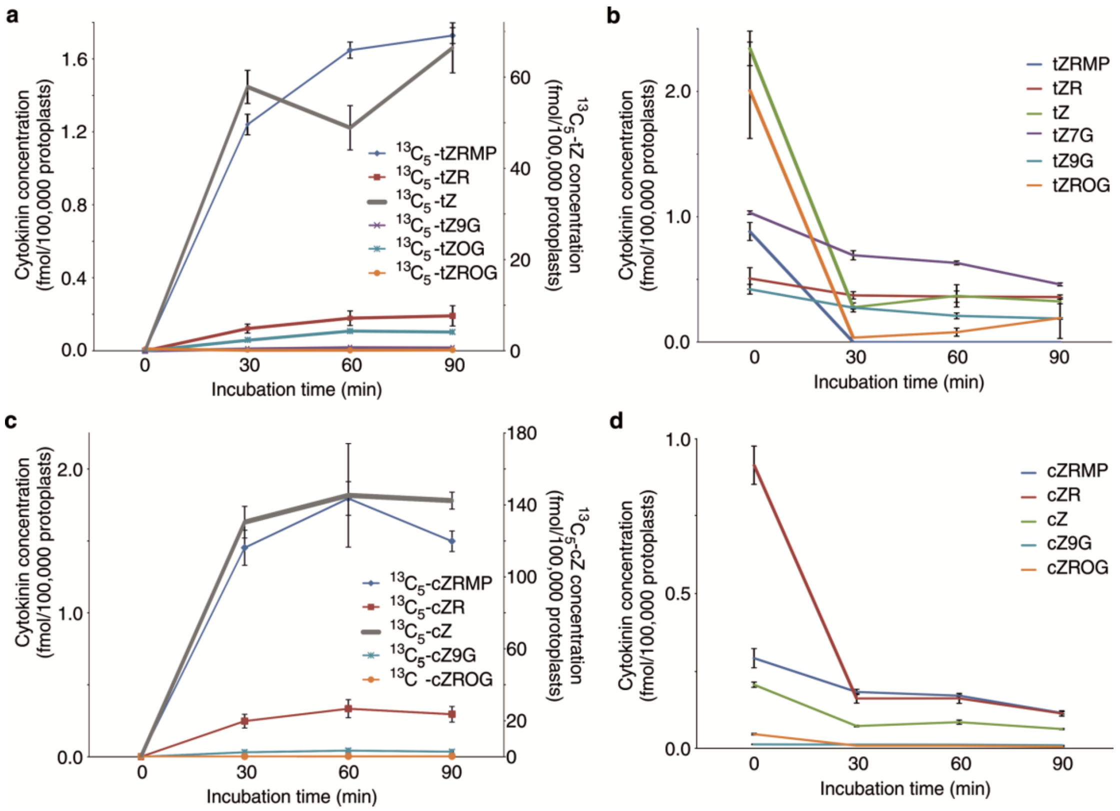


**Supplementary Figure 4. Uptake and metabolism of cytokinins by root protoplasts.** Root protoplast suspensions supplied with 1 µM stable isotope-labelled $^{13}C_5$-*t*Z (**a** and **b**) or $^{13}C_5$-*c*Z (**c** and **d**). **a,c** Major labelled metabolites detected by LCMS. Lefthand scale is for products, righthand scale is $^{13}C$ substrate. **b,d** Corresponding levels of endogenous (unlabelled) metabolites. Protoplast samples derived from 9-day-old Col-0 *Arabidopsis* seedling roots. Error bars rare s.e.m. While inactive forms ($^{13}C_5$-*t*ZRMP and $^{13}C_5$-*c*ZRMP, respectively) were the main metabolites in both treated protoplast samples (**a,c**), concentrations of most detected endogenous *t*Z- and *c*Z-cytokinins reduced over time, especially during the first 30 min after treatment (**b,d**). This suggests a tight homeostatic mechanism regulation on cytokinin metabolism with key components including the inactivation of active free bases (*t*Z and *c*Z) by conversion to cytokinin nucleotides, probably catalysed by APT enzymes.

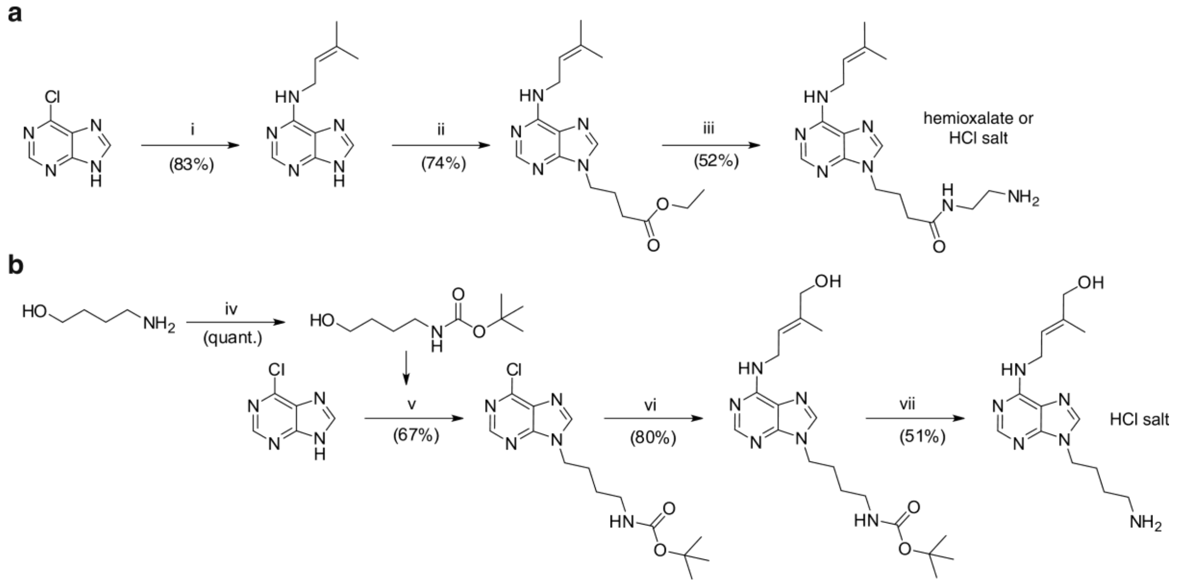


**Supplementary Figure 5. Reaction scheme for preparation of cytokinin ligands with linkers, used for immobilisation on pre-activated chromatography beads. a** Synthesis of iP-type ligand: i) 1.2 eq. 3-methylbut-2-en-1-amine hydrochloride, 2.5 eq. triethylamine, propanol, reflux, 4 h; ii) 1.1 eq. ethyl 4-bromobutyrate, 2.5 eq. $K_2CO_3$, N,N-dimethylformamide, 16 h; iii) 1/ 20 eq. ethane-1,2-diamine, reflux, 3 h; 2/ oxalic acid, methanol or ethanolic HCl, methanol, 0 °C. **b** Synthesis of *t*Z-type ligand: iv) 1 eq. Boc anhydride, 3 eq. $NaHCO_3$, tetrahydrofuran, methanol, 0 °C then room temperature, 16 h; v) 1.1 eq. tert-butyl (4-hydroxybutyl)carbamate, 2 eq. triphenylphosphine, 2 eq. diisopropyl azodicarboxylate, tetrahydrofuran, 2 h; vi) 1.3 eq. (E)-4-amino-2-methylbut-2-en-1-ol hemioxalate, 13 eq. triethylamine, methanol, 90 °C, 6 h; vii) 1/ Dowex 50W X8, dichloromethane, reflux, 6 h; 2/ 4 M methanolic $NH_3$, 16 h; 3/ ethanolic HCl, methanol, 0 °C. Reaction yields are shown in brackets. Original ChemDraw file is available on request.

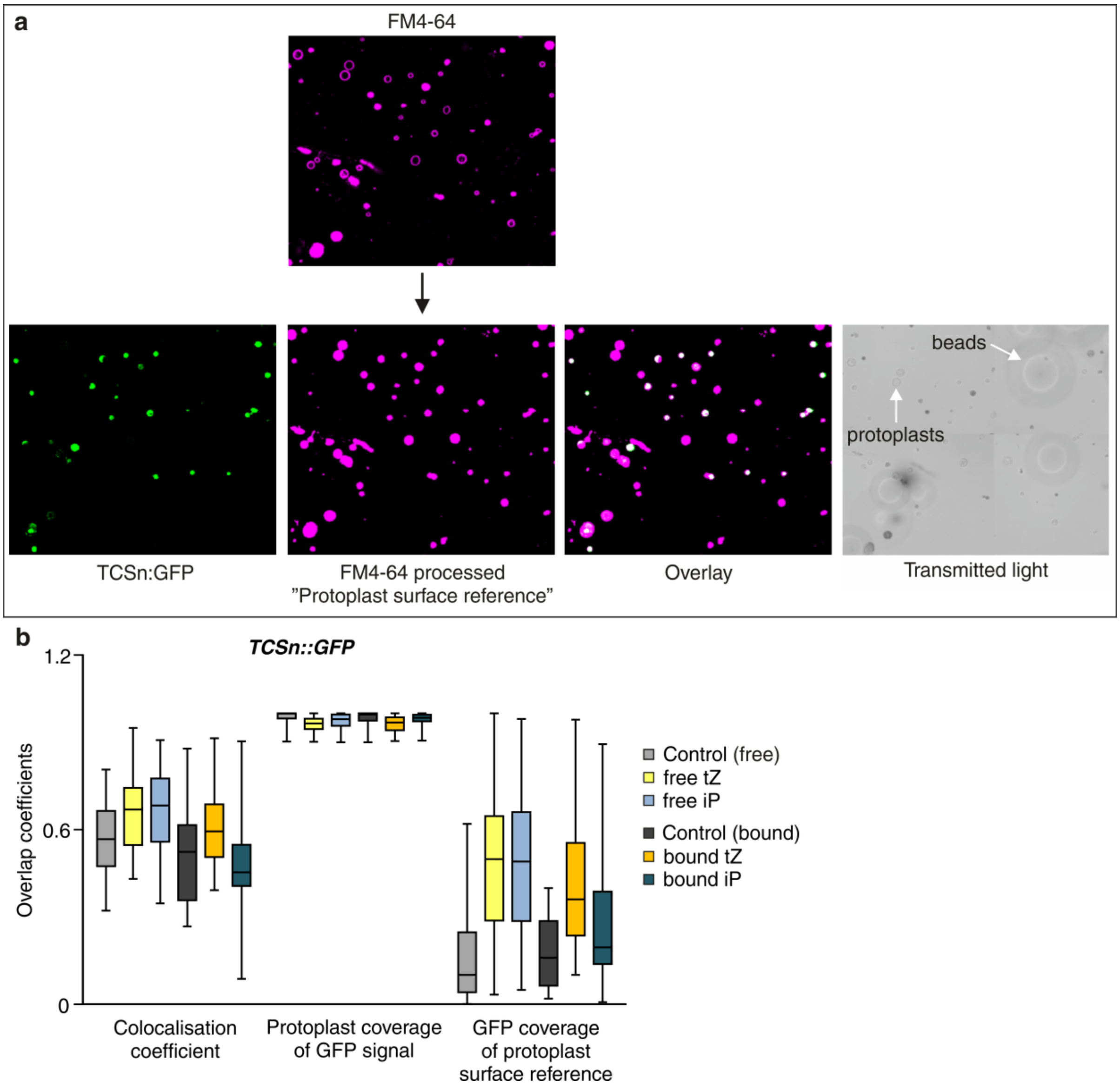


**Supplementary Figure 6. Quantification of GFP fluorescence intensity in root protoplasts.** **a** Illustration of image channels and overlay with indication of image processing method. **b** Quantification results for *TCSn::GFP* protoplasts treated overnight (~16 h) with 2 µM free iP or *tZ* or with membrane impermeable iP or *tZ* (ligand mean density 10 µmol $L^{-1}$) linked to Sepharose beads (denoted "bound", also referred to as extracellular compounds). Whiskers represent entire range of values, boxes indicate first and third quartiles, and central line is median

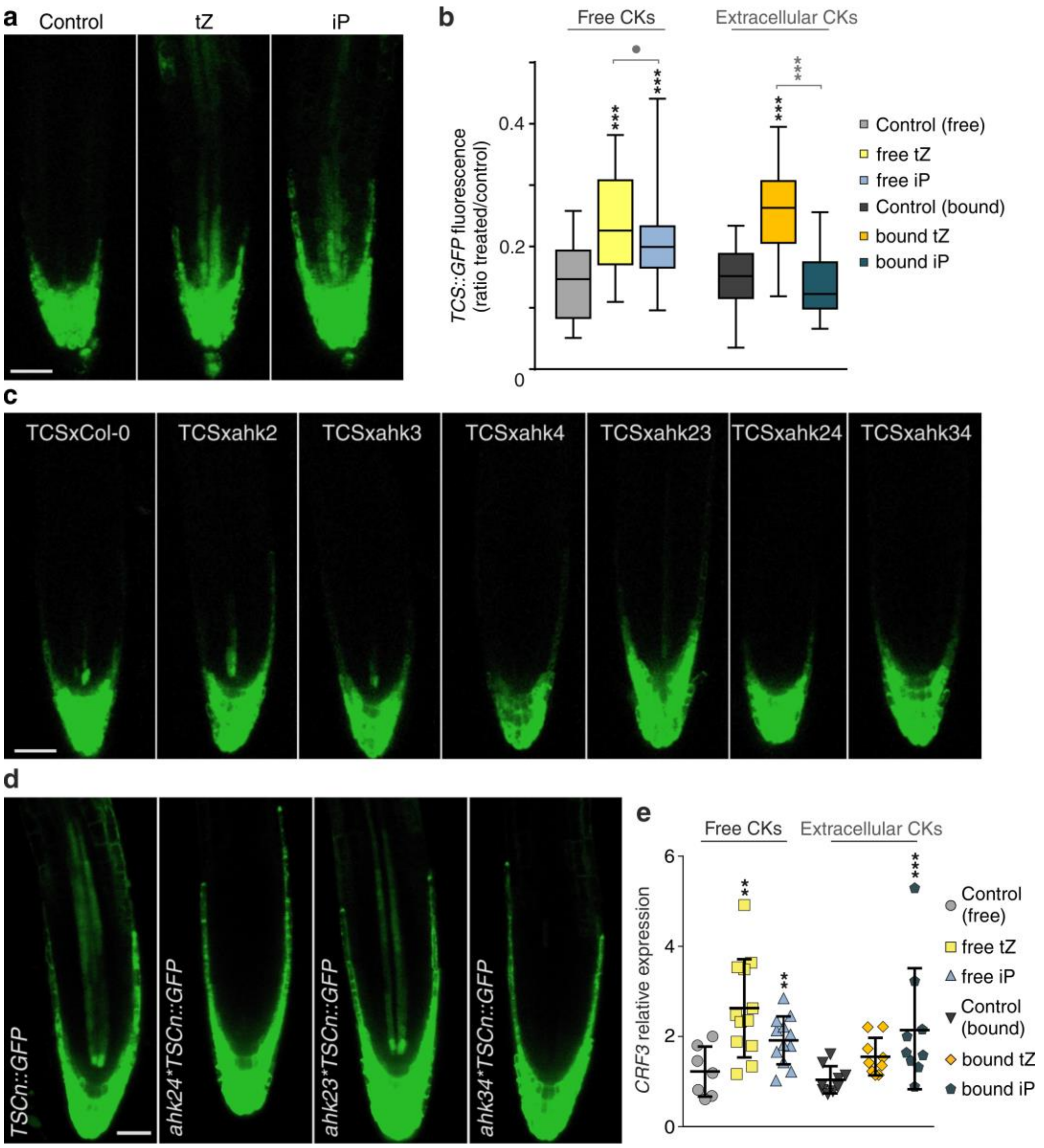


**Supplementary Figure 7. Cytokinin signalling responses to *t*Z and iP addition and to receptor mutations. a** *TCS::GFP* response 16 h after treatment with 2 µM iP or *t*Z. **b** Quantification of GFP fluorescence in protoplasts, derived from roots of 6 day-old *TCS::GFP* seedlings, after treatment with or without free cytokinins (*t*Z or iP, 2 µM) or immobilised cytokinins (*t*Z- or iP attached to Sepharose beads [ligand mean density 10 µmol $L^{-1}$], denoted "bound", also referred to as extracellular compounds). Whiskers represent entire range of values, boxes indicate first and third quartiles and central line is median; dot, P <0.1; ***, P <0.001 by one-way ANOVA, and Tukey's test indicating significant differences in fluorescence intensity between control and corresponding free or extracellular cytokinin treatments. Cytokinin treatment was applied for 16 h and 1 µM FM4-64 was added 10 min prior to confocal imaging. Each plot represents n>8 images, corresponding to >500 protoplasts. Scale bars 39 µm. Cytokinin treatment of *TCS::GFP* seedlings (**a**) and protoplasts (**b**) resulted in similar response trends to *TCSn::GFP* in corresponding experiments (Fig. 3a, b). However, fluorescence intensities in *TCS::GFP* mock samples and in response to cytokinin treatments were lower than those observed with *TCSn::GFP*. **c** TCS expression patterns in *ahk* mutant backgrounds. The *TCS::GFP* signal in stele cells is almost abolished when *AHK4* is mutated (*ahk4*, *ahk2,4, ahk3,4* mutants). **d** Confocal images of 6-day-old roots comparing *TCSn::GFP* expression in wild-type (Col-0), *ahk2,4,*

*ahk2,3* and *ahk3,4* backgrounds. **e** qRT-PCR analysis of the relative expression of *CYTOKININ RESPONSE FACTOR 3* (*CRF3*) induced by free and extracellular bound cytokinins. Dots are individual values, whiskers are s.d. and central line is mean, plotted as relative expression using the $2^{-\Delta\Delta Ct}$ method. Asterisks indicate values significantly different from the corresponding mock treatment in a Mann-Whitney *U* test (**, *P* <0.01; ***, *P* <0.001). Four biological replicates were analyzed in triplicate.

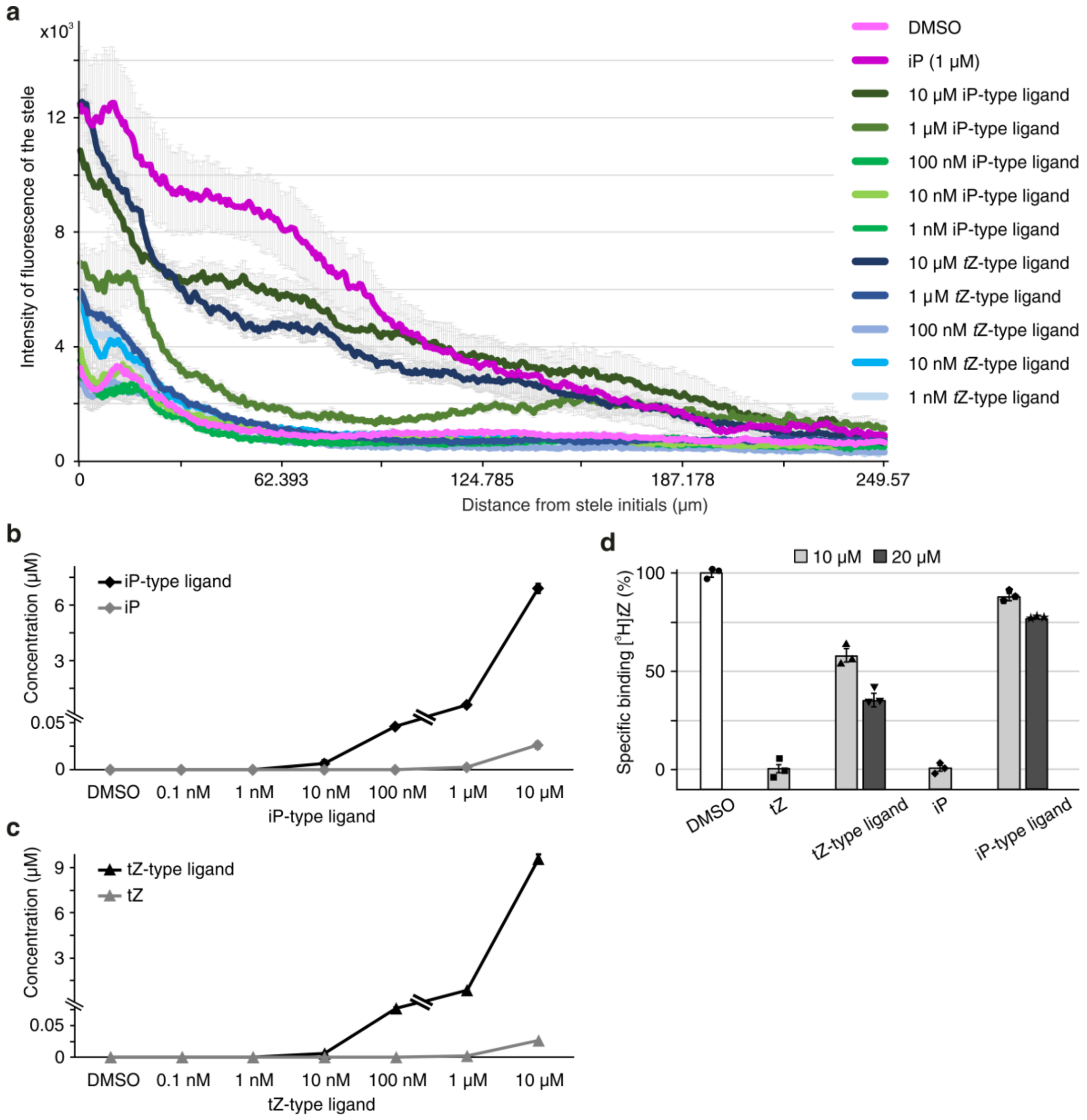


**Supplementary Figure 8. Control experiments on *t*Z- and iP-type ligands with attached linkers.** **a** Dose-response of relative GFP intensity in root tips of 6-day-old *TCSn::GFP* expressing seedlings treated with 1, 10, 100, $10^3$ or $10^4$ nM *t*Z- or iP-type ligands with linkers (compounds depicted in Supplementary Fig. 5), or $10^3$ nM free iP for 16 h. GFP fluorescence was quantified using ImageJ. DMSO was used as mock treatment. n≥4 roots imaged per treatment, error bars are s.e.m. The ligands with linkers can induce cytokinin response but are less active than free iP. **b** Quantification of free iP and iP-linker ligand in the remaining liquid media samples after the 16 h treatment from the experiment described in **a**. **c** Quantification of free *t*Z and *t*Z-linker ligand in the remaining liquid media samples after the 16 h treatment from the experiment described in **a**. **d** Competitive binding assay with heterologously expressed CRE1/AHK4 cytokinin receptor. Binding of 10 or 20 μM *t*Z-linker and iP-linker ligands were quantified in presence of 3 nM [2-$^3$H]*t*Z. Unlabeled iP and *t*Z (10 μM) were used as positive controls and 0.1% DMSO was used as a negative control. Dots are individual values, bar is mean with s.e.m.), n=3 replicates per treatment.

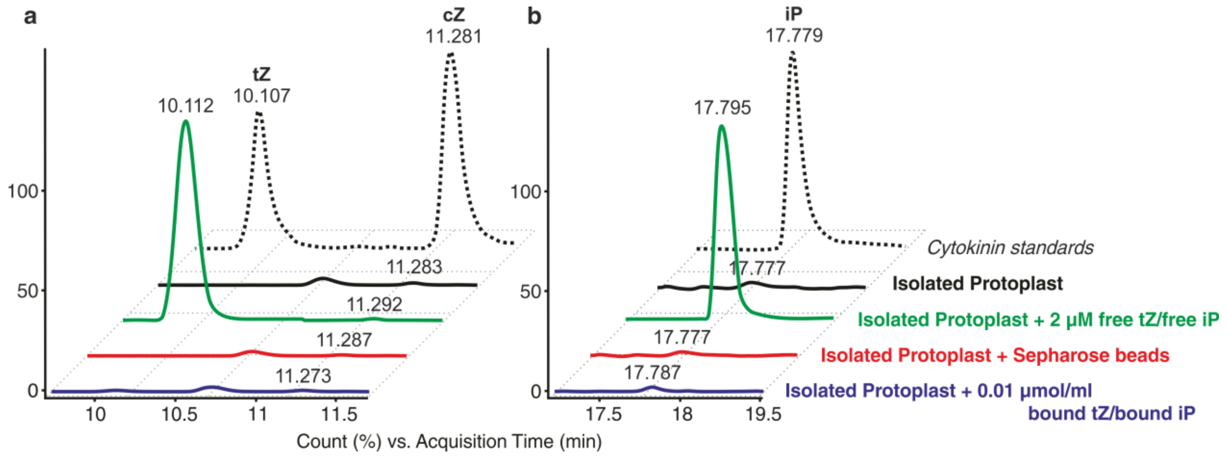


**Supplementary Figure 9. Sepharose-cytokinin beads stability tests.** The possibility that some free *t*Z (**a**) or iP (**b**) might be released from immobilised cytokinins (*t*Z or iP attached to Sepharose beads [ligand mean density 10 µmol $L^{-1}$], denoted "bound"), was examined by LC-MS/MS. The samples analysed were the same samples that were used for confocal imaging (Fig. 3a-c). The untreated protoplast profiles indicate low levels of endogenous cytokinins, whereas those treated with free cytokinins (2 µM) showed uptake of substantial excess of cytokinins. In contrast, incubation of protoplast with cytokinin beads resulted in minimal additional internal cytokinin.

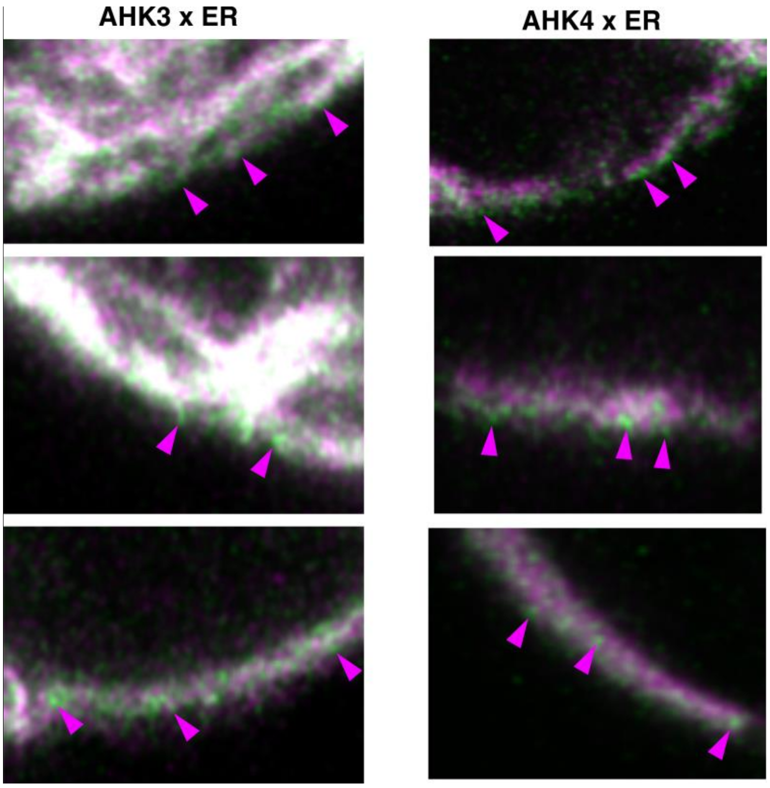


**Supplementary Figure 10. Additional zoomed-in 3D AiryScan images of AHK-GFP localisation in protoplasts.** AHK-GFP, green; ER marker RFP-p24δ5, magenta. Arrow heads point to cell surface localised AHK signals. Other details as in Fig. 4c.

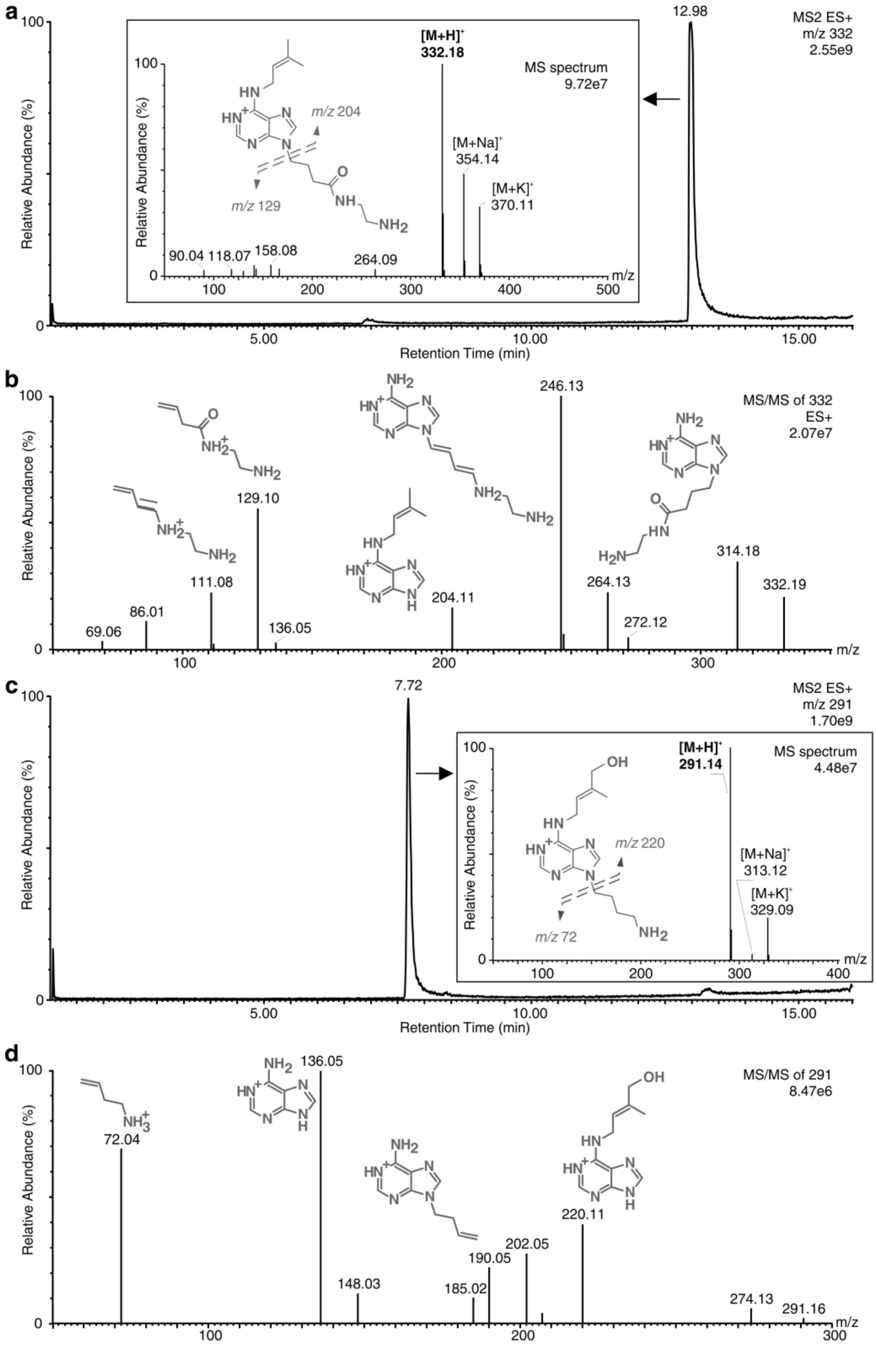


**Supplementary Figure 11. Mass spectral data of the cytokinin ligands.** Representative extracted ion chromatograms with full-scan (**a, c**) and product ion (**b, d**) mass spectra of the synthesised cytokinin ligands with linkers, obtained by UHPLC-ESI(+)-MS/MS. MS and MS/MS spectra of iP-type (*m/z* 332; **a, b**) and tZ-type (*m/z* 291; **c ,d**) ligands were analysed with a tandem mass spectrometer Xevo TQ-S in positive ion mode using collision energy 20 eV.

## SUPPLEMENTARY METHODS

**Chemical identity of new cytokinin ligands.** iP and *t*Z ligands possessing short linkers at the N9 position were synthesised in the Department of Chemical Biology and Genetics, Centre of the Region Haná for Biotechnological and Agricultural Research, Czech Republic according to the scheme shown in Supplementary Fig. 5. NMR spectra were recorded on a Jeol ECA-500 spectrometer operating at frequency 500 MHz ($^1H$) and 125 MHz ($^{13}C$), respectively. Samples were dissolved in DMSO-$d_6$ and the chemical shifts (δ) reported in ppm were calibrated to residual solvent peak (2.49 ppm for proton - DMSO-$d_5$, and DMSO-$d_6$ (39.5 ppm for carbon). The signals were assigned using 2D experiments such as COSY, HMBC, HMQC and in the spectra two tautomeric forms of products were observed. When the signals were separated, they were designated as form I and form II. High-performance liquid chromatography–UV–diode array–mass spectrometry (HPLC–UV–DAD–MS) experiments were performed as described[1]. Full MS and MS/MS characteristics are shown in Supplementary Fig. 11. For accurate identification of the major fragments generated by ESI(+)-MS/MS, a CFM-ID 3.0 program was used to predict the MS/MS fragmentation spectra for a given chemical structure[2]. Importantly, free cytokinin bases (iP and *t*Z) were present at <0.01% as possible impurities in both new iP and *t*Z ligands, as measured by UHPLC-MS/MS[3].

**iP-type ligand:** HPLC-UV-DAD (retention time, 13.05 min; purity, 99.99%), ESI$^+$-MS m/z (intensity 100%, ion M+H$^+$, 332.2). Two tautomeric forms in the ratio 2:1. $^1H$-NMR (500 MHz, DMSO-$d_6$) δ (ppm): 1.70 (s, 3H, $-NHCH_2CHC(C\underline{H}_3)_2$), 1.71 (s, 3H, $-NHCH_2CHC(C\underline{H}_3)_2$), 2.07 (q, *J* = 6.5 Hz, 2H, $-CH_2C\underline{H}_2CH_2CONH$), 2.12 (t, *J* = 6.6 Hz, 2H, $-CH_2CH_2C\underline{H}_2CONH$), 2.81 (td, *J* = 11.8, 6.0 Hz, 2H, $-CONHC\underline{H}_2CH_2NH_3^+$), 3.25 (q, *J* = 6.0 Hz, 2H, $-CONHCH_2C\underline{H}_2NH_3^+$), 4.19 (s, 1H, form I $-NHC\underline{H}_2CHC(C\underline{H}_3)_2$), 4.26 (s, 2H, $-C\underline{H}_2CH_2CH_2CONH$), 4.64 (s, 1H, form II $-NHC\underline{H}_2CHC(C\underline{H}_3)_2$), 5.35 (s, 1H, $-NHCH_2C\underline{H}C(CH_3)_2$), 8.11 (bs, 3H, $-CONHCH_2CH_2N\underline{H}_3^+$), 8.21 (t, *J* = 5.5 Hz, 1H, $-CH_2CH_2CH_2CON\underline{H}$), 8.46 (s, 1H, form I pur H2), 8.54 (s, 1H, form II pur H2), 8.56 (s, 1H, form II pur H8), 8.63 (s, 1H, form I pur H8), 9.50 (s, 1H, form II, $-N\underline{H}CH_2CHC(CH_3)_2$), 9.81 (bs, 1H, form I $-N\underline{H}CH_2CHC(CH_3)_2$). $^{13}C$-NMR (125 MHz, DMSO-$d_6$) δ (ppm): 18.1 ($-NHCH_2CHC(\underline{C}H_3)_2$), 25.2 ($-CH_2\underline{C}H_2CH_2CONH$), 25.4 ($-NHCH_2CHC(\underline{C}H_3)_2$), 31.9 ($-CH_2CH_2\underline{C}H_2CONH$), 36.4 ($-CONH\underline{C}H_2CH_2NH_3^+$), 38.5 ($-CONHCH_2\underline{C}H_2NH_3^+$), 39.5 (form I $-NH\underline{C}H_2CHC(CH_3)_2$), 41.5 (form II $-NH\underline{C}H_2CHC(CH_3)_2$), 43.3 (form II $-\underline{C}H_2CH_2CH_2CONH$), 43.5 (form I $-\underline{C}H_2CH_2CH_2CONH$), 117.5 (form I pur C5), 117.7 (form II pur C5), 118.9 ($-NHCH_2\underline{C}HC(CH_3)_2$), 119.6 ($-NHCH_2\underline{C}HC(CH_3)_2$), 136.1 (form I $-NHCH_2CH\underline{C}(CH_3)_2$), 136.3 (form II $-NHCH_2CH\underline{C}(CH_3)_2$), 142.9 (form I pur C8), 143.8 (form II pur C8), 144.8 (form II pur C2), 146.1 (form I pur C2), 146.8 (form I pur C4), 148.7 (form I pur C6), 149.1 (form II pur C6), 149.7 (form II pur C4), 171.63 ($-\underline{C}ONHCH_2CH_2NH_3^+$).

***t*Z-type ligand:** *HPLC-UV-DAD* (retention time, 11.10 min; purity, 99.99%), *ESI$^+$-MS m/z* (intensity 100%, ion M+H$^+$, 291.1). *Two tautomeric form in the ratio 1:1. $^1H$-NMR (500 MHz, DMSO-$d_6$) δ (ppm)*: 1.51-1.57 (m, 2H, $-CH_2CH_2C\underline{H}_2CH_2NH_3^+$), 1.66 (s, 3H, $-NHCH_2CHC(C\underline{H}_3)CH_2OH$), 1.86-1.92 (m, 2H, $-CH_2C\underline{H}_2CH_2CH_2NH_3^+$), 2.74-2.80 (m, 2H, $-CH_2CH_2CH_2C\underline{H}_2NH_3^+$), 3.79 (s, 2H, $-NHCH_2CHC(CH_3)C\underline{H}_2OH$), 4.29 (s, 3H, $-C\underline{H}_2CH_2CH_2CH_2NH_3^+$, form I $-NHC\underline{H}_2CHC(CH_3)CH_2OH$), 4.70 (s, 1H, form II $-NHC\underline{H}_2CHC(CH_3)CH_2OH$), 5.56 (s, 1H, $-NHCH_2C\underline{H}C(CH_3)CH_2OH$), 8.24 (s, 3H, $-CH_2CH_2CH_2CH_2N\underline{H}_3^+$), 8.45 (s, 1H, form I pur H2), 8.56 (s, 1H, form II pur H2), 8.64 (s, 1H, form II pur H8), 8.74 (s, 1H, form I pur H8), 9.67 (bs, 1H, form II $-N\underline{H}CH_2CHC(CH_3)CH_2OH$), 9.88 (bs, 1H, form I $-N\underline{H}CH_2CHC(CH_3)CH_2OH$). *$^{13}C$-NMR (125 MHz, DMSO-$d_6$) δ (ppm)*: 14.3 (form II $-NHCH_2CHC(\underline{C}H_3)CH_2OH$), 14.4 (form I $-NHCH_2CHC(\underline{C}H_3)CH_2OH$), 24.3 ($-CH_2CH_2\underline{C}H_2CH_2NH_3^+$), 26.8 ($-CH_2\underline{C}H_2CH_2CH_2NH_3^+$), 38.4 ($-CH_2CH_2CH_2\underline{C}H_2NH_3^+$), 39.4 (form I ($-NH\underline{C}H_2CHC(CH_3)CH_2OH$), 41.6 (form II $-NH\underline{C}H_2CHC(CH_3)CH_2OH$), 43.5

(form II $-\underline{C}H_2CH_2CH_2CH_2NH_3^+$), 43.8 (form I $-\underline{C}H_2CH_2CH_2CH_2NH_3^+$), 65.8 (form I $-NHCH_2CHC(CH_3)\underline{C}H_2OH$), 65.9 (form II $-NHCH_2CHC(CH_3)\underline{C}H_2OH$), 117.6 (form I pur C5), 117.7 (2× C, form I $-NHCH_2\underline{C}HC(CH_3)CH_2OH$), form II pur C5) 118.1 (form II $-NHCH_2\underline{C}HC(CH_3)CH_2OH$), 140.6 (form I $-NHCH_2CH\underline{C}(CH_3)CH_2OH$), 140.8 ($-NHCH_2CH\underline{C}(CH_3)CH_2OH$), 143.4 (form I pur C8), 144.4 (form II pur C8), 145.2 (form I pur C2), 146.9 (form I pur C4), 147.0 (for II pur C2), 148.7 (form I pur C6), 149.1 (for II pur C6), 149.5 (form II pur C4).

**Generation of cytokinins attached to Sepharose beads.** NHS-activated Sepharose™ 4 Fast Flow beads (GE Healthcare, United Kingdom) were washed with a threefold volume of anhydrous DMSO and resuspended in equal volume of 20 mM solution of cytokinin ligand in DMSO. Coupling was performed at room temperature under continuous moderate stirring overnight. Subsequently the beads were washed in three successive steps with DMSO, DMSO:water (1:1, v/v) and water, and remaining non-reacted *N*-hydroxysuccinimide groups were inactivated with 1 M ethanolamine according to the manufacturer's instructions. The cytokinin-coupled beads were rinsed then with water and ethanol:water (1:4, v/v) and stored in the latter solvent at 4°C until required. Control beads blocked with ethanolamine were prepared in the same way, omitting the ligand immobilisation step. To determine the efficiency of the coupling reaction, an aliquot of the cytokinin-coupled beads was thoroughly resuspended in glycerol:water (1:1, v/v) and its absorbance was measured at 272 nm. From this value, the absorbance of the blank beads was subtracted and the concentration of the immobilised cytokinin ligand was calculated following Lambert-Beer's law. Ligand density in undiluted packed beads was 2 mmol $L^{-1}$, and after dilution of the Sepharose beads, the final mean density of immobilised cytokinin ligands used was 10 or 5 µmol $L^{-1}$.

**Activation of *TCSn::GFP* response by iP-type and *t*Z-type ligands.** Six-day-old *TCSn::GFP* seedlings were transferred into 6-well plates containing 2 ml of standard Murashige and Skoog liquid medium with additions of 0.1% DMSO, 1 µM free iP, iP-type ligand or *t*Z-type ligand in increasing concentrations (1 nM, 10 nM, 100 nM, 1 µM or 10 µM). After 16 h, the treated seedlings were used for confocal imaging as described in *Methods* while the media that they were incubated in was collected for UHPLC-MS/MS analysis described below.

**Degradation of cytokinin-linker ligands to free cytokinins.** The media from the samples described above were collected and processed for cytokinin purification[4]. The purification process included additional samples derived from 1 ml standard Murashige and Skoog liquid medium after 0 h and 16 h incubation with 5 µmol $L^{-1}$ mean ligand density of iP or *t*Z attached to Sepharose beads. For quantification of cytokinin-linker ligands and their associated free bases, the samples were dissolved in 50 µl 10% methanol, 5 µl was injected onto a reversed-phase column (Acquity UPLC® Shield PR18 Column, 2.1 × 150 mm) and analyzed by UHPLC-MS/MS. The concentrations of iP and *t*Z were detected using method described[3]. Quantification of the cytokinin-linker ligands was obtained using a multiple reaction monitoring mode of $[M-H]^+$ and the appropriate product ion of free cytokinins (Supplementary Fig. 11), 332.1>204.1 and 291.1>220.1 for iP-type and *t*Z-type linker ligands, respectively. Optimal MS conditions were similar to the cytokinin profiling method[3], using collision energy 15 eV. The limits of detection (signal-to-noise ratio of 1:3) for all analytes were close to 0.5 fmol. The concentration range was at least over 4 orders of magnitude with a correlation coefficient 0.9994. Detachment of free cytokinin and cytokinin-linker ligands from Sepharose beads is reported in

Fig. 3c and Supplementary Table 1. Similarly, to monitor degradation of cytokinin-linker ligands to free cytokinins, two independent experiments were performed, reported in Supplementary Fig. 8b, c.

**Cytokinin ligand binding assay** Cytokinin standards were obtained from Olchemim (Olomouc, Czech Republic). Highly labelled trans-[2-$^3$H]zeatin ($^3$H-*t*Z; radiochemical purity >99%) was obtained from the Isotope laboratory of the Institute of Experimental Botany (Prague, Czech Republic). The binding assay was performed following the technique previously described[5,6] and using E. coli strain carrying the vector pINIIIΔEH expressing CRE1/AHK[7][1]. An aliquot of 1 ml of the E. coli suspension was transferred to Eppendorf tube and mixed with 1 µl cytokinin standard or cytokinin-linker ligand solution in DMSO (ligand concentration after dilution = 10 µM). As a negative control, 1 µl of DMSO instead of cytokinin was used. Subsequently, 2 µl of 1.5 µM $^3$H-tZ was added to every aliquot (concentration after dilution = 3 nM). The unlabeled cytokinin standards were used in high excess to discriminate between specific and non-specific binding. The mixture was vortexed and incubated for 30 minutes at 4 °C. Next, the sample was centrifuged (6000 *g*, 6 min, 4 °C) and the supernatant was removed using a vacuum pump. The pellet was thoroughly resuspended in 1 ml of scintillation cocktail (Beckman, Ramsey, MN, USA). Extracted radioactivity was measured with a Hidex 300 SL scintillation counter (Hidex, Finland) for 10 min for each sample. The experiment was performed with three independent replicates. and mean values with standard deviations were calculated.

## SUPPLEMENTARY REFERENCES


1. Buček, J., et al. Total synthesis of [$^{15}$N]-labelled C6-substituted purines from [$^{15}$N]-formamide – easy preparation of isotopically-labelled cytokinins and derivatives. *Royal Soc. Open Sci.* **5**, 181322 (2018).
2. Allen, F., Pon, A., Wilson, M., Greiner, R., Wishart, D. CFM-ID: a web server for annotation, spectrum prediction and metabolite identification from tandem mass spectra. *Nucleic Acids Res.***42**, 94–99 (2014).
3. Svačinová, J. et al. A new approach for cytokinin isolation from Arabidopsis tissues using miniaturized purification: pipette tip solid-phase extraction. *Plant Methods* **8**, 17 (2012).
4. Antoniadi, I. et al. Cell-type-specific cytokinin distribution within the Arabidopsis primary root apex. *Plant Cell* **27**, 1955–1967 (2015).
5. Romanov, G. A., Lomin, S. N. & Schmülling, T. Biochemical characteristics and ligand-binding properties of Arabidopsis cytokinin receptor AHK3 compared to CRE1/AHK4 as revealed by a direct binding assay. *J. Exp. Bot.* **57**, 4051–4058 (2006).
6. Kuderová, A. et al. Identification of AHK2- and AHK3-like cytokinin receptors in *Brassica napus* reveals two subfamilies of AHK2 orthologues. *J. Exp. Bot.* **66**, 339-353 (2015).
7. Suzuki, T. et al. The Arabidopsis sensor His-kinase, AHK4, can respond to cytokinins. *Plant Cell Physiol.* **42**, 107-114 (2001).
