# Supplementary material for: Cell-surface receptors enable perception of extracellular cytokinins
Source: Nat Commun. 2020 Aug 27;11:4284. doi: 10.1038/s41467-020-17700-9 (PMC7453015; doi:10.1038/s41467-020-17700-9)
Supplement: Supplementary file 3 — Reporting Summary [file 41467_2020_17700_MOESM3_ESM.pdf]

nature research

Corresponding author(s): Colin Turnbull, Karin Ljung

Last updated by author(s): Jul 3, 2020

Reporting Summary

Nature Research wishes to improve the reproducibility of the work that we publish. This form provides structure for consistency and transparency in reporting. For further information on Nature Research policies, seeAuthors & Referees and theEditorial Policy Checklist .

Statistics

For all statistical analyses, confirm that the following items are present in the figure legend, table legend, main text, or Methods section.

n/a	Confirmed	
☐	☒	The exact sample size (*n*) for each experimental group/condition, given as a discrete number and unit of measurement
☐	☒	A statement on whether measurements were taken from distinct samples or whether the same sample was measured repeatedly
☐	☒	The statistical test(s) used AND whether they are one- or two-sided<br>*Only common tests should be described solely by name; describe more complex techniques in the Methods section.*
☒	☐	A description of all covariates tested
☒	☐	A description of any assumptions or corrections, such as tests of normality and adjustment for multiple comparisons
☐	☒	A full description of the statistical parameters including central tendency (e.g. means) or other basic estimates (e.g. regression coefficient) AND variation (e.g. standard deviation) or associated estimates of uncertainty (e.g. confidence intervals)
☐	☒	For null hypothesis testing, the test statistic (e.g. *F*, *t*, *r*) with confidence intervals, effect sizes, degrees of freedom and *P* value noted<br>*Give P values as exact values whenever suitable.*
☒	☐	For Bayesian analysis, information on the choice of priors and Markov chain Monte Carlo settings
☒	☐	For hierarchical and complex designs, identification of the appropriate level for tests and full reporting of outcomes
☒	☐	Estimates of effect sizes (e.g. Cohen's *d*, Pearson's *r*), indicating how they were calculated

Our web collection on statistics for biologists contains articles on many of the points above.

Software and code

Policy information about availability of computer code

Data collection	All software is commercial or publicly available. FACS data collected BD FACSDiva version 6.1.2; LCMS data collected by Agilent software; confocal image data by Zeiss software on Zeiss LSM780. A custom MatLab script used for AiryScan image processing is available on request
Data analysis	All software is commercial or publicly available. Generic data processing done in Excel. FACS data analysed by BD FACSDiva version 6.1.2; LCMS data analysed by Mass Hunter software (version B.05.02; Agilent Technologies); confocal fluorescence quantification done with ImageJ modules as described in Methods

For manuscripts utilizing custom algorithms or software that are central to the research but not yet described in published literature, software must be made available to editors/reviewers. We strongly encourage code deposition in a community repository (e.g. GitHub). See the Nature Research guidelines for submitting code & software for further information.

Data

Policy information about availability of data

All manuscripts must include a data availability statement. This statement should provide the following information, where applicable:

- Accession codes, unique identifiers, or web links for publicly available datasets
- A list of figures that have associated raw data
- A description of any restrictions on data availability

No large datasets created and no accessions deposited in the course of this research; all plant lines and raw data sets available from corresponding authors on request

Field-specific reporting

Please select the one below that is the best fit for your research. If you are not sure, read the appropriate sections before making your selection.

- [x] Life sciences
- [ ] Behavioural & social sciences
- [ ] Ecological, evolutionary & environmental sciences

For a reference copy of the document with all sections, see nature.com/documents/nr-reporting-summary-flat.pdf

Life sciences study design

All studies must disclose on these points even when the disclosure is negative.

Sample size	Sample sizes were estimated as described in Figure legends and/or Methods. For sorting, collected sample protoplast numbers were reported by the instrument. For confocal-protoplast experiments a lower limit of 100 protoplasts was analysed, and the signal strength was compared between mock and treated sample. For apoplast/symplast sample size was determined as tissue fresh weight
Data exclusions	No data sets were excluded from the analyses reported. Individual values are provided on FACS plots and as dot plots overlaid on bar charts
Replication	Most experiments were done as 3 or more independent experiments on different days. For sorting experiments every biological replicate is also an independent experimental day. The only exception was the apoplast/symplast experiment that was done twice
Randomization	All samples were numbered and randomized before cytokinin purification and MS measurements. For quantification of TCSn signal in seedlings, folders containing the images were renamed. Likewise for quantification of TCSn signal in protoplast images after different treatments.
Blinding	The original identity of samples for MS was not shown as samples were labelled with numbers during data acquisition and analysis. Likewise for quantification of TCSn signal in protoplasts and seedlings. However during cell sorting and confocal imaging it was not possible to perform blinding.

Reporting for specific materials, systems and methods

We require information from authors about some types of materials, experimental systems and methods used in many studies. Here, indicate whether each material, system or method listed is relevant to your study. If you are not sure if a list item applies to your research, read the appropriate section before selecting a response.

Materials & experimental systems

n/a	Involved in the study	
x		Antibodies
x		Eukaryotic cell lines
x		Palaeontology
x		Animals and other organisms
x		Human research participants
x		Clinical data

Methods

n/a	Involved in the study	
x		ChIP-seq
	x	Flow cytometry
x		MRI-based neuroimaging

Flow Cytometry

Plots

Confirm that:

- [x] The axis labels state the marker and fluorochrome used (e.g. CD4-FITC).
- [x] The axis scales are clearly visible. Include numbers along axes only for bottom left plot of group (a 'group' is an analysis of identical markers).
- [ ] All plots are contour plots with outliers or pseudocolor plots.
- [x] A numerical value for number of cells or percentage (with statistics) is provided.

Methodology

Sample preparation	Arabidopsis TCSn::GFP plants and other mutant lines derived from TCSn::GFP; used to generate root protoplasts as per Methods
Instrument	BD FACS Aria I flow cytometer (BD Biosciences)
Software	BD FACSDiva version 6.1.2

Cell population abundance	50000 or 100000 sorting events per independent sample
Gating strategy	The initial gate (P1) was designed in a FSC-A/SSC-A bi-plot. The population selected as P1 was a clearly distinct population of large events (FSC-A>220.000). Then the P1 population was projected in the bi-plot of GFP – FITC and Autofluorescence – PE-Texas Red. The separation of populations into GFP negative and GFP positive was clear and both populations were positive in PE-Texas Red (>10^3). The boxes in Fig. 1a indicate the outputs of the gating strategy and no separate figure is provided in Supplementary Information.

☐ Tick this box to confirm that a figure exemplifying the gating strategy is provided in the Supplementary Information.
